# The predictive value of lymphocyte-to-monocyte ratio in the prognosis of acute coronary syndrome patients: a systematic review and meta-analysis

**DOI:** 10.1186/s12872-020-01614-x

**Published:** 2020-07-15

**Authors:** Xiao-Qing Quan, Run-Chang Wang, Qing Zhang, Cun-Tai Zhang, Lei Sun

**Affiliations:** 1 Department of General Practice, Shenzhen Longhua District Central Hospital, Shenzhen, 518110 China; 2 Second clinical medical college, Tongji Medical College, Huazhong University of Science and Technology, Wuhan, 430030 China; 3 Department of Neurology, Tongji Hospital, Tongji Medical College, Huazhong University of Science and Technology, Wuhan, 430030 China; 4 Department of Geriatrics, Tongji Hospital, Tongji Medical College, Huazhong University of Science and Technology, Wuhan, 430030 China; 5 Department of Pathology, Zhujiang Hospital, Southern Medical University, 253 Gongye Road, Guangzhou, 510282 China

**Keywords:** Lymphocyte-to-monocyte ratio, Mortality, Major adverse cardiac events, Acute coronary syndrome

## Abstract

**Background:**

The association between the lymphocyte-to-monocyte ratio (LMR) and prognosis in the patients with acute coronary syndrome (ACS) is not fully understood. We performed this systematic review and meta-analysis to evaluate the correlation between LMR and mortality or major adverse cardiac events (MACE) in patients with ACS.

**Methods:**

A systematic search was performed in PubMed, MEDLINE, EMBASE, the Cochrane Library, Scopus, and Web of science. The association between LMR and mortality/MACE was analyzed in patients with ACS. The search was updated to April 15, 2020.

**Results:**

A total of 5 studies comprising 4343 patients were included in this meta-analysis. The results showed that lower LMR predicted higher short-term mortality/MACE (hazard ratio [HR] = 3.44, 95% confidence interval [CI]: 1.46–8.14, *P* <  0.05) and long-term mortality/MACE (HR = 1.70, 95% CI: 1.36–2.13, *P* <  0.05). In the subgroup analysis, there was still statistical significance of long-term mortality/MACE in all subgroups.

**Conclusions:**

This study suggested that lower LMR value might be associated with higher short-term and long-term mortality/MACE in ACS patients. Especially for younger ACS patients, low LMR was more closely associated with poor prognosis.

## Background

Coronary heart disease (CHD) is one of the largest causes of death and disease burden worldwide [1, 2]. Acute coronary syndrome (ACS) is a severe category of CHD associated with a high morbidity and mortality. ACS includes unstable angina (UA), ST-segment elevation myocardial infarction (STEMI), and non-ST-segment elevation myocardial infarction (NSTEMI). Previous studies indicate that approximately half of deaths from CHD occur after ACS [3, 4]. Rupture of atherosclerotic plaques and formation of thrombi are the main cause of ACS [5–7]. The atherosclerotic plaques are associated with the infiltration of inflammatory cells (lymphocytes, monocytes, and neutrophils) [8–10]. Inflammation plays a critical role in initiation, progression, and rupture of atherosclerotic plaque in ACS patients [9, 10].

Markers of inflammation are associated with the prognosis of patients with ACS. The neutrophil-to-lymphocyte ratio (NLR) has been established as a valuable predictor of the prognosis of ACS [11–13]. Compared with neutrophils, monocytes play a more important role in the pathogenesis of atherosclerotic disease [14]. The role of monocyte infiltration of the arterial wall in the development of atherosclerotic plaques is well recognized [15]. In addition, previous studies have showed that monocytes are associated with the onset of myocardial infarction (MI) and left ventricular remodeling [16, 17].

In recent years, a growing body of research has focused on the relationship between lymphocyte-to-monocyte ratios (LMR) and mortality or major adverse cardiac events (MACE) in patients with ACS. However, the conclusions of these studies are controversial. For example, Gijsberts et al. indicated that LMR significantly improved prediction of mortality [18]. In the latter study, Kristono et al. found that LMR is not enough to be used for prediction in a clinical setting [19]. Herein, we performed this meta-analysis to explore the predictive value of LMR in ACS patients.

## Methods

This meta-analysis was performed followed the Preferred Reporting Items of Systematic Reviews and Meta-Analyses (PRISMA) statement. We registered this meta-analysis in the PROSPERO database (CRD42019131296).

### Search strategy

A systematic literature search was conducted in PubMed, MEDLINE, EMBASE, the Cochrane Library, Scopus, and Web of science. We used the following terms to search literature: “STEMI”,“UA”, “NSTEMI”, “lymphocyte to monocyte ratio”, “lymphocyte-to-monocyte ratio”, “lymphocyte/monocyte ratio”, “monocyte/lymphocyte ratio”, “mortality”, “MACE” and “major adverse cardiac events”. The latest update was performed in April 15, 2020. We also screened the reference lists of all retrieved articles to identify other potentially relevant literature.

### Inclusion and exclusion criteria

Studies were included if they met all the following criteria: (1) articles were published as full-text in English; (2) patients with ACS (STEMI, UA, NSTEMI); (3) LMR (hazard ratio [HR], 95% confidence interval [CI]) was available; (4) the outcomes were associated with mortality or MACE. Articles were excluded if they met any of the following characteristics: (1) nonhuman studies; (2) duplicate studies; (3) absence of LMR or mortality/MACE. Two investigators (Xiao-Qing Quan and Run-Chang Wang) read the literature independently of each other. Disagreements solved by discussion with other investigators.

### Data extraction and quality assessment

The following data were extracted: the first author, the country of patients, duration, the mean age, sample size of patients, LMR cut-off value, diseases of patients, HRs and 95% CIs and outcomes. The outcomes of studies included mortality (all-cause mortality) and MACE (including stroke/transient ischemic attack, target vessel revascularization, non-fatal MI, and cardiac death). The methodological quality of each study was evaluated with Newcastle-Ottawa Scale (NOS) system [20]. The maximum score is 9 and the study with a NOS score ≥ 6 was considered as a high-quality study.

### Statistical analysis

All statistical analyses in the present study were conducted with STATA statistical software (version 13.1, Stata Corporation, College Station, TX, USA). We synthesized the HR and corresponding 95% CI to analysis of the relationship between LMR and mortality/MACCE. Between-study heterogeneity was assessed using Cochrane’s Q and I^2^ texts. I^2^ < 25% was regarded as low levels of heterogeneity. I^2^ value of 25 to 50% was regarded as moderate levels of heterogeneity. I^2^ > 50% was regarded as high levels of heterogeneity. A fixed-effects model was applied in the absence of significant heterogeneity (I^2^ ≤ 50%), or the random effect model was applied (I^2^ > 50%).

## Results

### The literature search and include studies

**Fig. 1:**
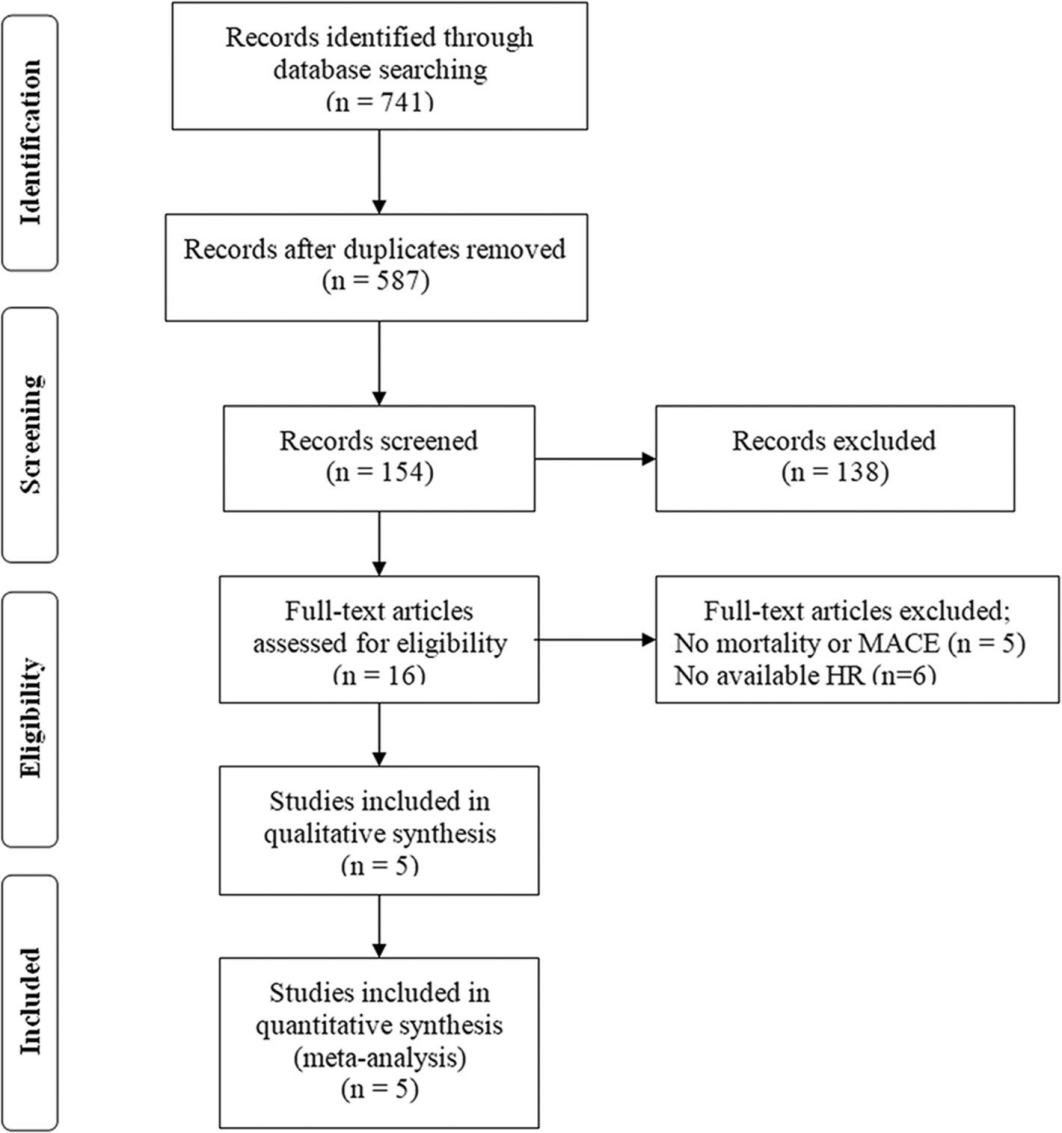
PRISMA flowchart describing the literature search and article selection

A flowchart of the literature search was shown in Fig. 1. Initially, in the primary search from the major databases, a total of 741 studies were included. After removing duplicates and screening titles and abstracts, a total of 154 papers remained, but 138 of them did not meet our purpose. The remaining 16 articles were assessed for eligibility based on full-text review, 11 were deemed ineligible. After qualitative and quantitative analysis, according to the inclusion criteria, only 5 studies published from 2016 to 2019 were selected for our meta-analysis [18, 21–24].

Basic characteristics of the included studies were listed in Table 1. A total of 4343 patients were included. These studies were all observation researchers and one conducted in Netherlands [18], one conducted in Turkey [21], three conducted in China [22–24]. The mean age of the patients ranges from 60.77 to 65.12 years old. Two studies in this meta-analysis enrolled STEMI patients [21, 24]. Two studies enrolled NSTEMI patients [22, 23], and the remaining one study enrolled ACS patients [18]. Two of studies explicitly stated that the patients underwent PCI [21, 22], while others did not specify if enrolled patients underwent PCI [18, 23, 24]. Two studies reported the mortality [18, 21], and three studies reported MACE [22–24]. All the studies have reported adjusted HR values. Adjusted confounding factors of each study were shown in Table 2. According to the Newcastle-Ottawa scale (NOS) [20], all cohort studies were of high quality and had scores of seven or more.

**Table 1:** The main characteristics of the included studies

Study (year)	Country	Duration	Mean Age (years)	LMR cut-off value	Patient’s diseases	Sample	Outcomes	Quality (NOS)
Gijsberts CM (2016) [18]	Netherlands	2010–2013	65.12	3.11	ACS	1015	Long-term mortality	8
Kiris T (2017) [21]	Turkey	2010–2013	61.5	1.67	STEMI	318	30-day mortality 36-month mortality	7
Fan Z (2018) [22]	China	2010–2015	62.34	2.78	NSTEMI	678	Long-term MACE	7
Cheng H (2019) [23]	China	2013–2017	60.77	2.33	NSTEMI	963	In-hospital MACE Long-term MACE	8
Cai M (2019) [24]	China	2014–2017	63.08	1.84	STEMI	1369	Long-term MACE	8

*Abbreviations*: *ACS* acute coronary syndrome, *LMR* lymphocyte-to-monocyte ratio, *MACE* major adverse cardiac events, *NSTEMI* non-ST-elevated myocardial infarction, *NOS* Newcastle-Ottawa scale, *STEMI* ST-elevated myocardial infarction

**Table 2:** HR and adjusted confounding factors of included studies

Study (year)	Outcomes	HR(95%CI)	Adjusted confounding factors
Gijsberts CM (2016) [18]	Long-term mortality	1.35 (1.14–1.59)	Leukocyte characteristics (lymphocyte cell size coefficient of variation, monocyte count)
Kiris T (2017) [21]	30-day mortality 36-month mortality	8.093 (1.006–65.074) 2.374 (1.160–4.857)	Age, gender, history of stroke/TIA, history of DM, multivessel disease, Killip, albumin, LVEF, hemoglobin, RDW, MPV, serum creatinine, total bilirubin, β-blocker usage, ACEI/ARB usage
Fan Z (2018) [22]	Long-term MACE	2.128 (1.458–3.105)	NLR, hs-CRP, brain natriuretic peptide
Cheng H (2019) [23]	In-hospital MACE Long-term MACE	2.891 (1.265–8.354) 1.793 (1.169–2.515)	Age, male, body mass index, hypertension, DM, dyslipidemia, history of coronary artery disease, history of myocardial infarction, smoking index, Leukocyte, NLR, hs-CRP, gensini score
Cai M (2019) [24]	Long-term MACE	1.74 (1.12–2.70)	Age, sex, Killip, DM, hypertension, hyperlipidemia, PCI, β-blocker usage, ACEI/ARB usage, glucose, white blood cell, hemoglobin, ln CK-peak, MPV, RDW, LVEF, location of myocardial infarction

*Abbreviations*: *ACEI* angiotensin-converting enzyme inhibitors, *ARB* angiotensin receptor blockers, *CI* confidence interval, *DM* diabetes mellitus, *HR* hazard ratio, *hs-CRP* high-sensitivity C reactive protein, *LVEF* left ventricular ejection fraction, *MPV* mean platelet volume, *NLR* neutrophil-to-lymphocyte ratio, *PCI* percutaneous coronary intervention, *RDW* red cell distribution width, *TIA* transient ischemic attack

### LMR and mortality/MACE

**Fig. 2:**
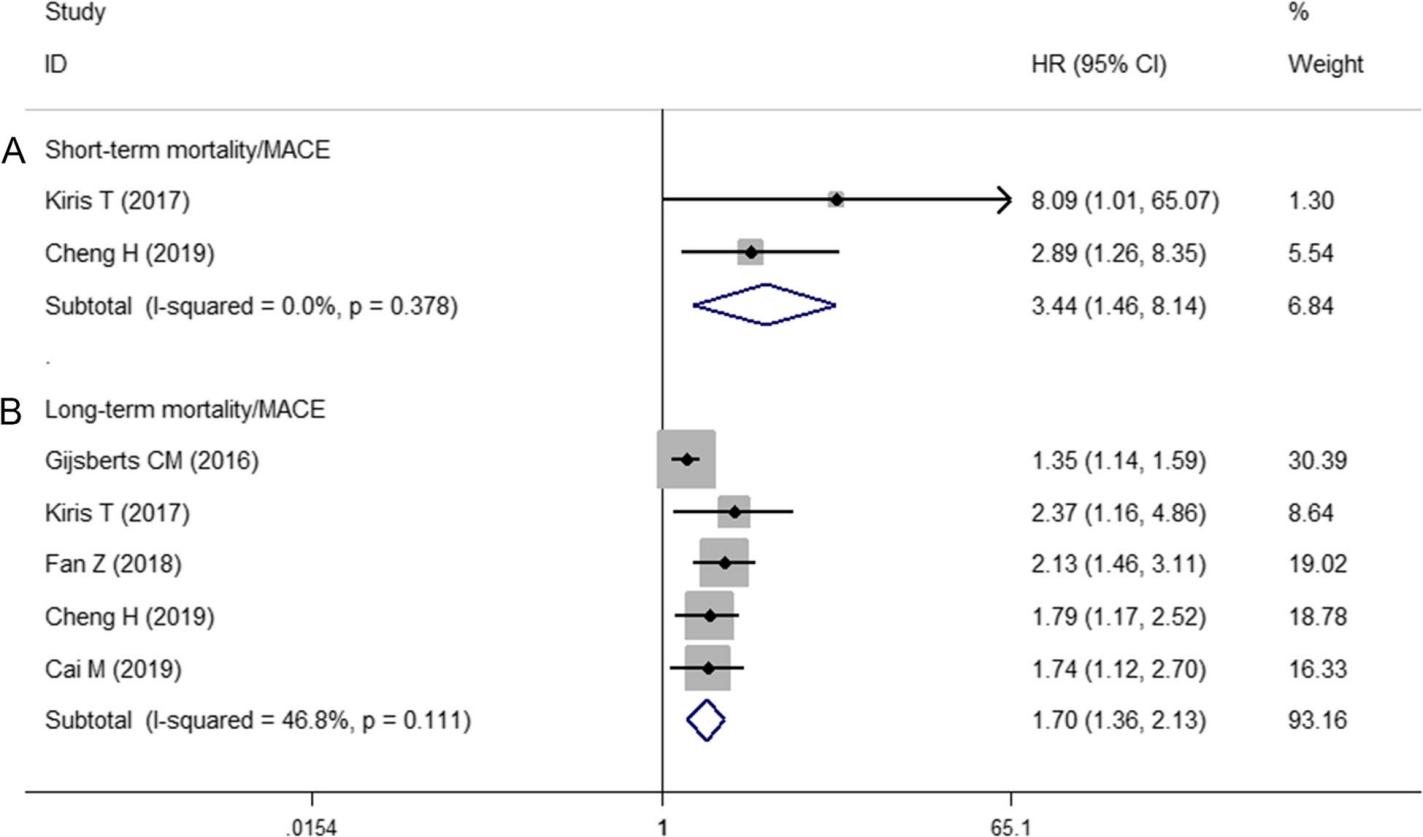
Forest plot of the association between LMR and outcomes. **a** Low LMR predicted short-term mortality/MACE. **b** Low LMR predicted long-term mortality/MACE. *CI* confidence interval, *HR* hazard ratio, *LMR* lymphocyte-to-monocyte ratio, *MACE* major adverse cardiac events

The short-term was defined as within 30 days after admission to hospital. If the hospitalization lasted more than 30 days, the excess was also included. Others were defined as long-term. The combined analysis of 2 studies covering 1281 patients described the relationship between LMR and short-term mortality/MACE [21, 23]. The result showed that LMR predicted short-term mortality/MACE (HR = 3.44, 95% CI: 1.46–8.14, *P* <  0.05, Fig. 2a), with low levels of heterogeneity among studies (I^2^ = 0%). The combined analysis of 5 studies covering 4343 patients described the relationship between LMR and long-term mortality/MACE [18, 21–24]. The pooled outcome for low LMR value compared with high LMR value group was found to be 1.70 (95% CI: 1.36–2.13, *P* <  0.05, Fig. 2b), with moderate levels of heterogeneity among studies (I^2^ = 46.8%).

### Subgroup analysis

There were moderate levels of heterogeneity (I^2^ = 46.8%) in the analysis of LMR predicting long-term mortality/MACE. We performed subgroup analysis according to mean age (≥ 62 and < 62), LMR cut-off value (≥ 2 and < 2), sample size (≥ 1000 and < 1000) and diseases of patients (ACS, STEMI and NSTEMI). The results were shown in Table 3. Compared with older ACS patients (≥ 62), LMR had better predictive value of long-term mortality/MACE in younger ACS patients (< 62). And low LMR predicted long-term mortality/MACE showed a statistical significance in any subgroup. Based on the change of I^2^, the sources of heterogeneity might be mean age of enrolled patients and defined cut-off value (Table 3). In the subgroup of older (≥ 62) ACS patients, I^2^ increased to 61.8%. In the subgroup of higher (≥ 2) LMR cut-off value, I^2^ increased to 64.7%.

**Table 3:** The association between LMR and long-term mortality/MACE according to different subgroups

Subgroup	Study (No.)	I^2^ (%)	P (I^2^)	HR (95% CI)	P (HR)
Mean Age					
≥ 62	3	61.8	0.073	1.64 (1.22, 2.21)	< 0.001
< 62	2	0	0.498	1.91 (1.36, 2.68)	< 0.001
Cut-off value					
≥ 2	3	64.7	0.059	1.66 (1.24, 2.23)	< 0.001
< 2	2	0	0.469	1.89 (1.30, 2.76)	< 0.001
Sample					
≥ 1000	2	10.6	0.290	1.41 (1.17, 1.69)	< 0.001
< 1000	3	0	0.728	2.00 (1.56, 2.58)	< 0.001
Disease					
ACS	1	NA	NA	1.35 (1.14, 1.59)	< 0.001
STEMI	2	0	0.469	1.89 (1.30, 2.76)	< 0.001
NSTEMI	2	0	0.533	1.96 (1.49, 2.54)	< 0.001

*Abbreviations*: *CI* confidence interval, *HR* hazard ratio, *NA* not applicable

## Discussion

ACS has a high morbidity and remains one of the major causes of mortality in the world [3, 4]. Previous studies have suggested that LMR may be associated with the prognosis of ACS patients [21–23, 25, 26]. Here we performed this meta-analysis to analyze the relationship between LMR and the prognosis of ACS patients. The aggregated results showed that a lower LMR might predict a higher mortality/MACE in patients with ACS.

In this meta-analysis, we enrolled 5 studies comprising 4343 patients to investigate the prognostic value of the LMR in patients with ACS [18, 21–24]. The present study showed that LMR might be a predictor for short-term mortality/MACE. However, only two studies examined the effect of LMR on short-term mortality/MACE. More related studies are needed to explore the predictive value of low LMR for short-term mortality.

Results from the present study suggested that lower LMR was associated with higher long-term mortality/MACE in patients with ACS. Because there was a moderate level of heterogeneity among studies, we conducted subgroup analysis to further analyze the results. In all subgroups, LMR still had predictive value for poor prognosis, which indicated that the results were relatively reliable. Meanwhile, we found that mean age and defined cut-off value might be the sources of heterogeneity. We hypothesized that older ACS patients had more complex factors affecting the prognosis, such as immune status and nutritional status, leading to higher inter-study heterogeneity. And higher cut-off value had worse predictive value for poor prognosis. This might be the reason that heterogeneity occurred among studies with higher cut-off value. Our results also showed that low LMR was valuable for predicting poor prognosis in STEMI and NSTEMI patients, which was consistent with previous researches [21–24].

ACS is related to atherosclerosis, which is accompanied by the infiltration of inflammatory cells [8–10]. Lymphocytes and monocytes are pivotal immune cells and play an important role in inflammatory response and atherosclerosis development [27, 28]. Previous studies indicated that decreased lymphocytes and increased monocytes might be related to the poor prognosis of the MI patients [29–31].. Lymphocytes might be driven by recognition of cardiac auto antigens, became activated after MI, and facilitated the healing of the myocardium [29]. MI could activate adrenergic signaling and trigger the production of monocytes. Excessive mononuclear growth might impair myocardial healing and exacerbate cardiovascular complications [30, 31]. The above results indicated that lymphocyte and monocyte might be related to the prognosis of the MI patients.

Our studies had some limitations. Firstly, we did subgroup analysis and identified possible sources of heterogeneity. We could not accurately locate heterogeneity because of the subgroup analysis was observational. Secondly, only five studies were included in the meta-analysis, potentially leading to heterogeneity and less persuasive. Thirdly, all the enrolled studies were observational researchers. Compared with experimental studies, observational studies are more likely to have the risk of bias, which also relatively influence the accuracy of the study.

To the best of our knowledge, this is the first meta-analysis addressing the relationship between LMR and the mortality/MACE in patients with ACS. This meta-analysis showed that LMR could be a valuable predictor in predicting mortality/MACE in patients with ACS. What’s more, in many primary hospitals, routine blood is the most rapid and basic detection methods which can immediately determine the patient’s condition. LMR might be used as an inexpensive and useful marker in assessment of patients with ACS.

## Conclusions

In summary, this meta-analysis showed that a low LMR value might be effective in predicting the risk of short-term and long-term mortality/MACE in patients with ACS. Especially for younger ACS patients, a low LMR value might be more effective in predicting poor prognosis. But additional research was required to verify its effectiveness.

## Data Availability

Because this is a meta-analysis, all of data included in this study could be found in the included references.

## Abbreviations

LMR: Lymphocyte-to-monocyte ratio
CHD: Coronary heart disease
ACS: Acute coronary syndrome
MACE: Major adverse cardiac events
HR: Hazard ratio
CI: Confidence interval
STEMI: ST-elevated myocardial infarction
UA: Unstable angina
NSTEMI: Non-ST-segment elevation myocardial infarction
NLR: Neutrophil-to-lymphocyte ratio
MI: Myocardial infarction
PRISMA: Preferred reporting items of systematic reviews and meta-analyses
NOS: Newcastle-Ottawa scale
PCI: Percutaneous coronary intervention
TIA: Transient ischemic attack
DM: Diabetes mellitus
LVEF: Left ventricular ejection fraction
RDW: Red cell distribution width
MPV: Mean platelet volume
ACEI: Angiotensin-converting enzyme inhibitors
ARB: Angiotensin receptor blockers
hs-CRP: High-sensitivity C reactive protein
